# TRIB2 desensitizes ferroptosis via βTrCP-mediated TFRC ubiquitiantion in liver cancer cells

**DOI:** 10.1038/s41420-021-00574-1

**Published:** 2021-07-27

**Authors:** Susu Guo, Yuxin Chen, Xiangfei Xue, Yueyue Yang, Yikun Wang, Shiyu Qiu, Jiangtao Cui, Xiao Zhang, Lifang Ma, Yongxia Qiao, Jiayi Wang

**Affiliations:** 1 Department of Clinical Laboratory, Shanghai Tenth People’s Hospital, Tongji University, Shanghai, 200072 China; 2 Department of Clinical Laboratory, West China Second University Hospital, Sichuan University, Sichuan, 610041 China; 3 Shanghai Institute of Thoracic Oncology, Shanghai Chest Hospital, Shanghai Jiao Tong University, Shanghai, 200030 China; 4 School of Public Health, Shanghai Jiao Tong University School of Medicine, Shanghai, 200025 China

**Keywords:** Targeted therapies, Oncogenes

## Abstract

Tribbles homolog 2 (TRIB2) is known to boost liver tumorigenesis via regulating Ubiquitin (Ub) proteasome system (UPS). At least two ways are involved, i.e., acts as an adaptor protein to modulate ubiquitination functions of certain ubiquitin E3 ligases (E3s) and reduces global Ub levels via increasing the proteolysis activity of proteasome. Recently, we have identified the role of TRIB2 to relieve oxidative damage via reducing the availability of Ub that is essential for the ubiquitination and subsequent degradation of Glutathione peroxidase 4 (GPX4). Although GPX4 is a critical antioxidant factor to protect against ferroptosis, the exact evidence showing that TRIB2 desensitizes ferroptosis is lacking. Also, whether such function is via E3 remains unclear. Here, we demonstrated that deletion of TRIB2 sensitized ferroptosis via lifting labile iron in liver cancer cells. By contrast, overexpression of TRIB2 led to the opposite outcome. We further demonstrated that transferrin receptor (TFRC) was required for TRIB2 to desensitize the cells to ferroptosis. Without TFRC, the labile iron pool could not be reduced by overexpressing TRIB2. We also found that beta-transducin repeat containing E3 ubiqutin protein ligase (βTrCP) was a genuine E3 for the ubiquitination of TFRC, and TRIB2 was unable to decline labile iron level once upon βTrCP was knocked out. In addition, we confirmed that the opposite effects on ferroptosis and ferroptosis-associated lipid reactive oxygen species (ROS) generation resulted from knockout and overexpression of TRIB2 were all indispensible of TFRC and βTrCP. Finally, we demonstrated that TRIB2 exclusively manipulated RSL3- and erastin-induced-ferroptosis independent of GPX4 and glutathione (GSH). In conclusion, we elucidated a novel role of TRIB2 to desensitize ferroptosis via E3 βTrCP, by which facilitates TFRC ubiquitiation and finally decreases labile iron in liver cancer cells.

## Introduction

TRIB2 is a pseudokinase that has a major function to act as a molecular adaptor mediating protein ubiquitination and followed destabilization via E3s [1]. An unique feature distinguishing TRIB2 from other genuine protein kinases is that TRIB2 has an atypical pseudokinase domain retaining a regulated binding platform for context-specific E3s to ubiquitinate substrates [1]. Vast majority of studies have also suggested that TRIB2 can interact with a serial of E3s, such as βTrCP [2], SMAD specific E3 ubiquitin protein ligase 1 (Smurf1, [3]) and COP1 E3 ubiquitin ligase (COP1, [4]) at its C terminus. Recently, the roles of TRIB2 are expanded by our study demonstrating that TRIB2 can modulate proteasome function for the reduction of global Ub and protection of liver cancer cells against oxidative stress [5]. However, whether TRIB2 has other functions to boost tumorigenesis in liver cancer cells is still not very clear.

TRIB2 has been revealed as one of the key molecules stabilizing GPX4, the critical hydroperoxidase capable of converting lipid hydroperoxides into non-toxic lipid alcohols, thereby relieving cell damage under oxidative stress [5]. Inhibition of GPX4 either at genetic or pharmacological level causes ferroptosis [6, 7]. Ferroptosis is defined as an iron-dependent regulated necrosis involving excessive lipid peroxidation, and is distinct from apoptosis, necrosis, and autophagy [8]. However, whether TRIB2 also modulates the sensitivity of cells to the induction of ferroptosis is not known thus far.

Ferroptosis and iron metabolism are intimately associated. Meanwhile, the imbalance of intracellular iron homeostasis is also a major cause for carcinogenesis [9]. Because of the important role of iron, its homeostasis is tightly regulated [10]. TFRC after binding with transferrin (TF) is sorted to endosomes, where the binding of TFRC, TF, and iron are dissolved in an acidic environment [11]. Fe (III) in endosomes is reduced prior to the release to cytosol via Solute carrier family 11 member 2 (SLC11A2, also known as DMT1), and Fe (II) is subsequently captured by iron chaperones poly (rC) binding protein 1/2 (PCBP1/2) and guided to its destination for functional use or storage in ferritin [12, 13]. Interestingly, ferritin-stored Fe (III) can be retrieved via Nuclear receptor coactivator 4 (NCOA4)-mediated ferritinophagy, an autophagy-associated specific degradation of ferritin to recover Fe (II) [12]. Emerging studies also demonstrated that the labile iron provided by ferritinophagy following induction of ferroptosis is essential for cells to sustain their ferroptosis sensitivity [14]. Recently, we revealed that TRIB2 interacts with poly(rC) binding protein 2 (PCBP2, [5]), thus we speculate that TRIB2 might be also involved in iron metabolism; however, whether TRIB2 has a role to regulate the sensitivity of cells to ferroptosis via iron metabolism is currently unknown.

In our present study, the effects and mechanisms by which TRIB2 modulates the sensitivity of cells to ferroptosis via iron metabolism were investigated. We demonstrated that TRIB2 acts as an adaptor protein, which is essential for E3 βTrCP-mediated TFRC ubiquitination and followed destabilization. Via such effects, labile iron is decreased to desensitize cells to ferroptosis. Given that TRIB2 is upregulated and pro-tumorigenic in liver cancer cells [3, 15], we thereby elucidate another role of TRIB2 to boost liver tumorigenesis via sustaining liver cancer cells in a ferroptosis-resistant state.

## Results

### TRIB2 desensitized liver cancer cells to ferroptosis

**Fig. 1:**
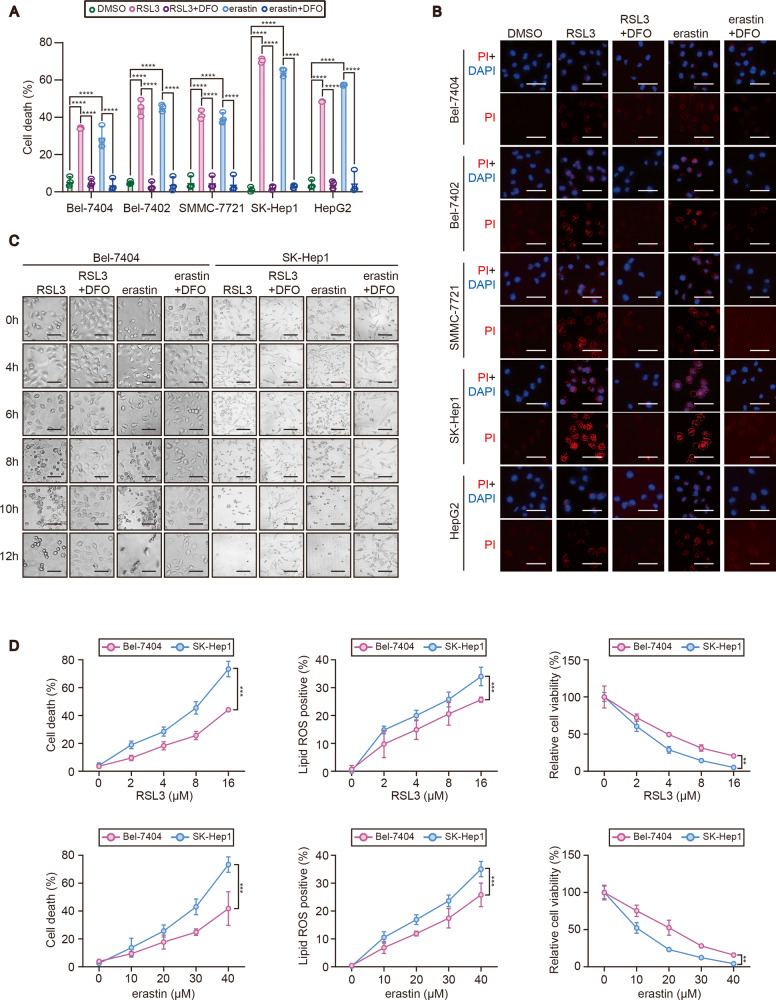
Sensitivity to ferroptosis was varied among liver cancer cells. **A** Percentage of death cells in liver cancer cells, as indicated following treating with RSL3 (5 µM, 12 h) or erastin (10 µM, 12 h) with or without DFO (25 µM, 12 h), as measured by staining with Sytox green following flow cytometry. **B** PI staining of liver cancer cells as indicated following treating with RSL3 (5 µM, 6 h) or erastin (10 µM, 6 h) with or without DFO (25 µM, 6 h). Scale bar, 50 μM. **C** Cell morphology changes of Bel-7404 and SK-Hep1 cells following treating with RSL3 (5 µM) or erastin (10 µM) with or without DFO (25 µM) for indicated hours. Scale bar, 50 μM. **D** Cell death, lipid ROS generation and cell viability in Bel-7404 and SK-Hep1 cells following treating with the indicated concentration of RSL3 or erastin for 12 h, as measured by Sytox green and C11-BODIPY staining following flow cytometry and a ATP detection kit. Data were analyzed by one-way ANOVA and expressed as mean ± SD from three independent experiments. NS non-significance; **P < 0.01; ***P < 0.001; ****P < 0.0001.

To investigate whether TRIB2 desensitizes liver cancer cells to ferroptosis, a serial of liver cancer cell lines including Bel-7404, Bel-7402, SMMC-7721, SK-Hep1, and HepG2 cells were tested for their sensitivity to RSL3 and erastin, two well-established ferroptosis agonists. We found a significant cell death in all the tested liver cancer cell lines following treating with either RSL3 or erastin for 12 h, and such effects could be prevented by simultaneously treating with iron chelator DFO (Fig. 1A), suggesting ferroptosis did occur. Results from staining of propidium iodide, which is assumed to permeate into the dead cells also demonstrated that most of liver cancer cell lines tested were prone to death following treating with erastin or RSL3 for 6 h (Fig. 1B). Combined with the results from Fig. 1A, B, we found that Bel-7404 had the least sensitivity, while SK-Hep1 had the most sensitivity for the induction of ferroptosis among the cell lines tested. Thereby, we chose the Bel-7404 and SK-Hep1 cells for the following studies. Overt ferroptosis began to occur at 8 h following treating with either RSL3 or erastin in Bel-7404 cells, while was 2 h earlier (~6 h) in SK-Hep1 cells (Fig. 1C), further demonstrating that SK-Hep1 cells are more sensitive to ferroptosis than Bel-7404 cells. We further treated Bel-7404 and SK-Hep1 cells with a serial concentration of RSL3 and erastin, and found that the degree of RSL3 and erastin-induced cell death, ferroptosis-associated lipid ROS generation, and reduced cell viability in SK-Hep1 cells were more obvious than those in Bel-7404 cells (Fig. 1D).

**Fig. 2:**
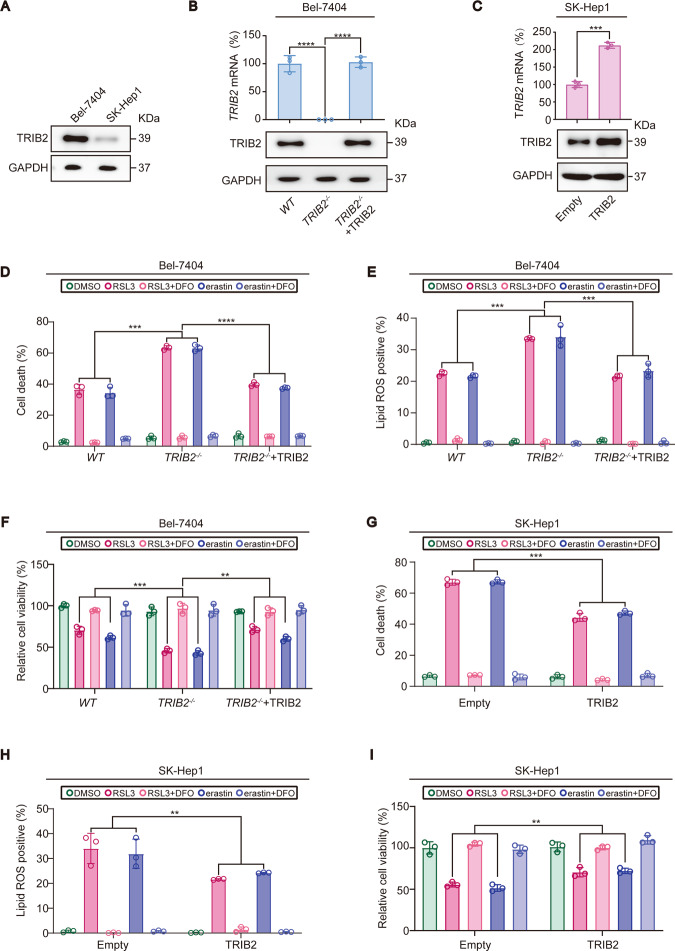
Sensitivity to ferroptosis was determined by TRIB2 in liver cancer cells. **A** Endogenous TRIB2 protein expression level in Bel-7404 and SK-Hep1 cells, as measured by IB. TRIB2 expression before and after TRIB2 was knocked out in Bel-7404 cells (**B**) and overexpressed in SK-Hep1 cells (**C**), as measured by qPCR and IB. TRIB2-sg resistant TRIB2 was also simultaneously overexpressed in *TRIB2*^−/−^ Bel-7404 cells for the rescue experiments. Cell death, lipid ROS generation, and cell viability were measured in Bel-7404 cell with TRIB2 knocked out in the presence or absence of TRIB2 reconstitution, or in SK-Hep1 cells with TRIB2 overexpressed following treating with RSL3 (5 µM, 12 h) or erastin (10 µM, 12 h) with or without DFO (25 µM, 12 h), as measured by Sytox green following flow cytometry (**D**, **G**), C11-BODIPY staining following flow cytometry (**E**, **H**) and a ATP detection kit (**F**, **I**). Data were analyzed by one-way ANOVA and expressed as mean ± SD from three independent experiments. NS non-significance; **P < 0.01; ***P < 0.001; ****P < 0.0001. Images of all the immunoblots are representative of three independent experiments.

Does the expression level of TRIB2 determine the degree of the sensitivity to the induction of ferroptosis in Bel-7404 and SK-Hep1 cells? To answer this, TRIB2 expression was evaluated in Bel-7404 and SK-Hep1 cells. We found that TRIB2 was expressed at a higher level in Bel-7404 cells than that in SK-Hep1 cells (Fig. 2A), suggesting that lower expression of TRIB2 in SK-Hep1 cells might be critical for higher sensitivity to the induction of ferroptosis. Because the expression of TRIB2 is higher in Bel-7404 cells than that in SK-Hep1 cells, TRIB2 was knocked out in Bel-7404 cells and overexpressed in SK-Hep1 cells, respectively to evaluate whether TRIB2 desensitizes cells to ferroptosis (Fig. 2B, C). In Bel-7404 cells, knocking TRIB2 out aggravated the induced ferroptosis-associated cell death and lipid ROS generation as well as reduced cell viability that is caused by RSL3 and erastin (Fig. 2D–F). Notably, such effects could be reversed by simultaneously expressing a TRIB2-sg-resistant TRIB2 (Fig. 2D–F). By contrast, overexpressing TRIB2 in SK-Hep1 cells mitigated the effects by RSL3 and erastin (Fig. 2G–I). Collectively, these results demonstrated that TRIB2 desensitizes liver cancer cells to ferroptosis.

### TRIB2 decreased labile iron level in liver cancer cells

**Fig. 3:**
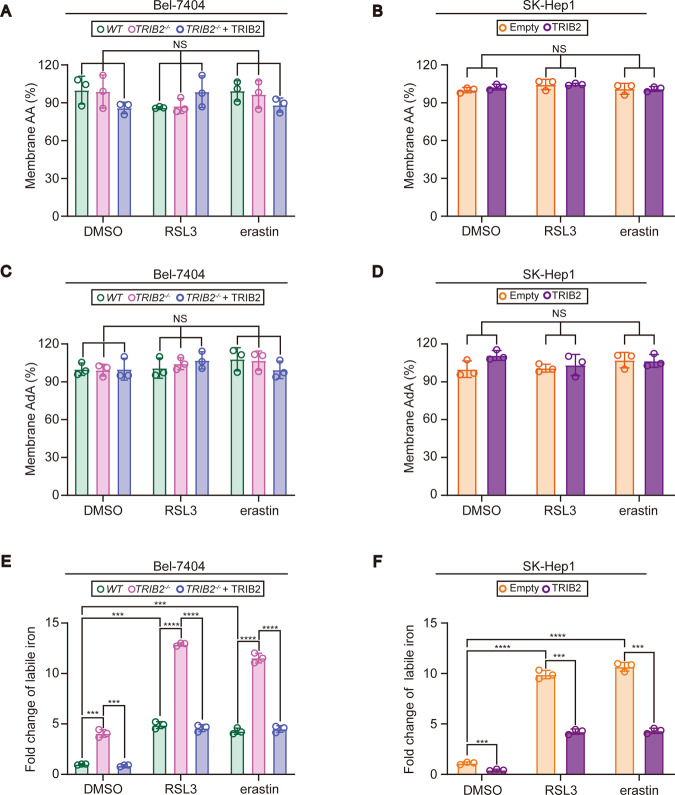
TRIB2 decreased labile iron level in liver cancer cells. AA and AdA in the membrane fractions were measured in Bel-7404 cells with TRIB2 knocked out with or without reconstitution of TRIB2 (**A**, **C**), and in SK-Hep1 cells with or without TRIB2 overexpression (**B**, **D**) in basal condition or following treating with RSL3 (5 µM, 12 h) or erastin (10 µM, 12 h). Membrane fractions were extracted by a Fatty Acid Extraction Kit, while AA and AdA were measured by ELISA-based kits. **E**, **F** TRIB2 decreased labile iron in liver cancer cells. Labile iron was measured in Bel-7404 and SK-Hep1 cells under the same conditions as those in **A**–**D**. Data were analyzed by one-way ANOVA and expressed as mean ± SD from three independent experiments. NS non-significance; ***P < 0.001; ****P < 0.0001.

During ferroptosis, toxic lipid ROS is accumulated as a result from the reaction between iron and lipid peroxides [16]. While lipid peroxides can be generated by the oxidation of polyunsaturated fatty acid (PUFA)-containing phospholipids, arachidonic acid (AA), and adrenic acid (AdA) are two major PUFAs that are oxygenated [17]. Because membranes from mitochondria, lysosomes, and endoplasmic reticulum are all sites of lipid ROS accumulation during ferroptosis [17, 18], membrane fractions were thereby extracted from Bel-7404 cells with or without TRIB2 knockout and SK-Hep1 cells with or without TRIB2 overexpression to investigate whether TRIB2 influences the levels of AA and AdA in the membrane. Unfortunately, AA and AdA in the membrane fractions could not be affected by TRIB2 following treating with RSL3 and erastin (Fig. 3A–D). However, knocking TRIB2 out in Bel-7404 cells remarkably boosted the basal and increased labile iron level induced by RSL3 and erastin, and these effects could be reversed by simultaneously overexpressing TRIB2 (Fig. 3E), suggesting that TRIB2 acts to mitigate labile iron. Such conclusion was further supported by the observation that overexpressing TRIB2 decreased labile iron at basal level and also the one induced by RSL3 and erastin in SK-Hep1 cells (Fig. 3F).

### TFRC was essential for TRIB2 to reduce labile iron in liver cancer cells

**Fig. 4:**
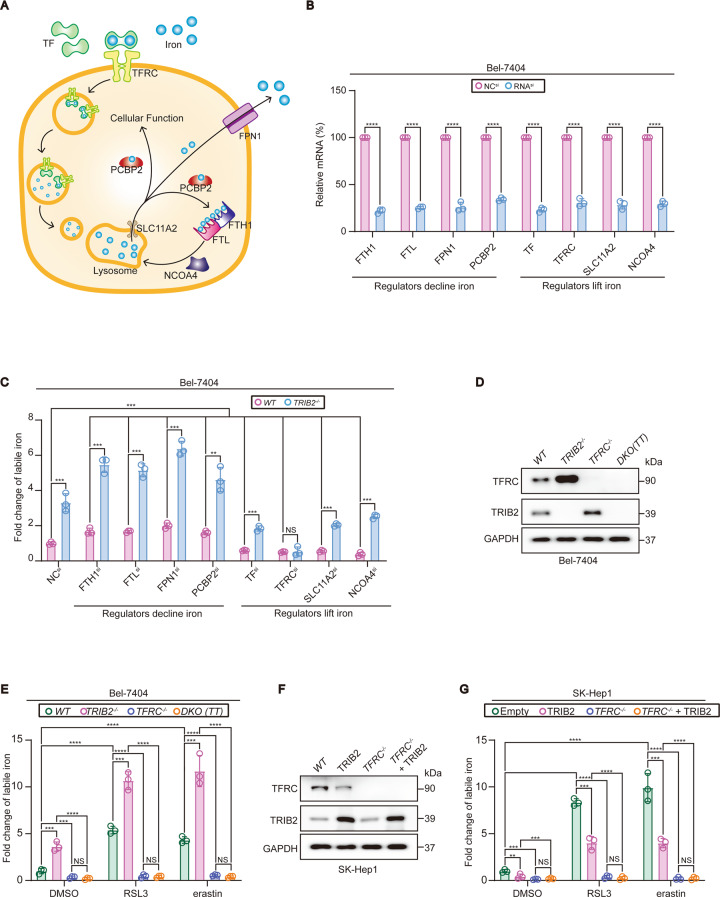
TRIB2 reduced labile iron level in a TFRC-dependent manner. **A** Schematic presentation of iron metabolism. **B** mRNA levels of indicated iron regulators before and after knocking down by siRNA, as measured by qPCR. **C** Deletion of TRIB2 elevated labile iron via TFRC in Bel-7404 cells. Labile iron was measured by Iron Assay Kit before and after TRIB2 was knocked out in Bel-7404 cells with siRNA-mediated knockdown for indicated iron regulators. Deletion of TRIB2 elevated labile iron via TFRC. TFRC and TRIB2 were measured by IB (**D**). Labile iron was measured by Iron Assay Kit (**E**) in Bel-7404 cells with or without single or double knockout (DKO) of TRIB2 and TFRC in basal condition or following treating with RSL3 (5 µM, 12 h) or erastin (10 µM, 12 h). **F**, **G** Overexpression of TRIB2 reduced labile iron also via TFRC. TFRC and TRIB2 were measured by IB (**F**). Labile iron was measured by Iron Assay Kit (**G**) before and after overexpressing TRIB2 in SK-Hep1 cells with or without TFRC knocked out in basal condition or following treating with RSL3 (5 µM, 12 h) or erastin (10 µM, 12 h). Data were analyzed by Student’s *t* test and expressed as mean ± SD from three independent experiments. NS non-significance; **P < 0.01; ***P < 0.001; ****P < 0.0001. Images of all the immunoblots are representative of three independent experiments.

Next, we investigated how TRIB2 reduces labile iron in liver cancer cells. Iron absorption, transfer, storage, and retrieval are tightly regulated by numerous mechanisms [19]. Here, we investigated the functions of a serial of iron regulators during the process in which TRIB2 reduces labile iron. Ferritin heavy chain 1 (FTH1) and ferritin light chain (FTL) are heavy and light chain of ferritin consisting 24 subunits, which is critical for iron storage [20]. FPN1 is the major factor to export iron from cytoplasm into the blood flow [21]. In addition, PCBP2 was identified as one of the major iron chaperones that guide iron to its destination for functional use or storage in ferritin [13]. Recently, we revealed that PCBP2 is also positively regulated by TRIB2 ([5]). While FTH1, FTL, FPN1, and PCBP2 are critical to reduce intracellular iron, TF, TFRC, and SCL11A2 are critical to absorb and transport iron into the cytoplasm [12, 13, 20–23]. Besides, NCOA4 is also essential to increase intracellular iron because it is required for ferritinophagy, a ferritin-specific autophagy to take iron out of ferritin [24]. Given that these factors function to either decline or lift intracellular iron level (illustrated in Fig. 4A), we knocked down these factors and examined whether TRIB2 reduces iron levels in a way dependent on these factors. Firstly, the knockdown efficiencies of siRNAs targeting against these iron-associated factors were verified in Bel-7404 cells (Fig. 4B). Indeed, knocking FTH1, FTL, FPN1, and PCBP2 down in Bel-7404 cells significantly lifted while knocking TF, TFRC, SLC11A2, and NCOA4 down significantly declined labile iron level (Fig. 4C). TRIB2 deletion still increased labile iron level even when FTH1, FTL, FPN1, PCBP2, TF, SLC11A2, and NCOA4 was knocked down in Bel-7404 cells; however, the effects were diminished when TFRC was knocked down (Fig. 4C), suggesting that TRIB2 reduces labile iron via TFRC.

To further examine whether TFRC is critical for TRIB2 to reduce labile iron in basal condition or during ferroptosis, TFRC was further knocked out by CRISPR/Cas9 technology in Bel-7404 and SK-Hep1 cells treated with or without RSL3 and erastin. We found that TRIB2 knockout had no effects to increase labile iron level when TFRC was deleted in Bel-7404 cells both in basal condition and following treating with RSL3 and erastin (Fig. 4D, E). On the contrary, TRIB2 overexpression in SK-Hep1 cells was also unable to mitigate labile iron in the absence of TFRC (Fig. 4F, G). These results demonstrated that TFRC is essential for TRIB2 to reduce labile iron both in basal condition and during ferroptosis.

### βTrCP-mediated ubiquitination of TFRC was essential for TRIB2 to decline labile iron

**Fig. 5:**
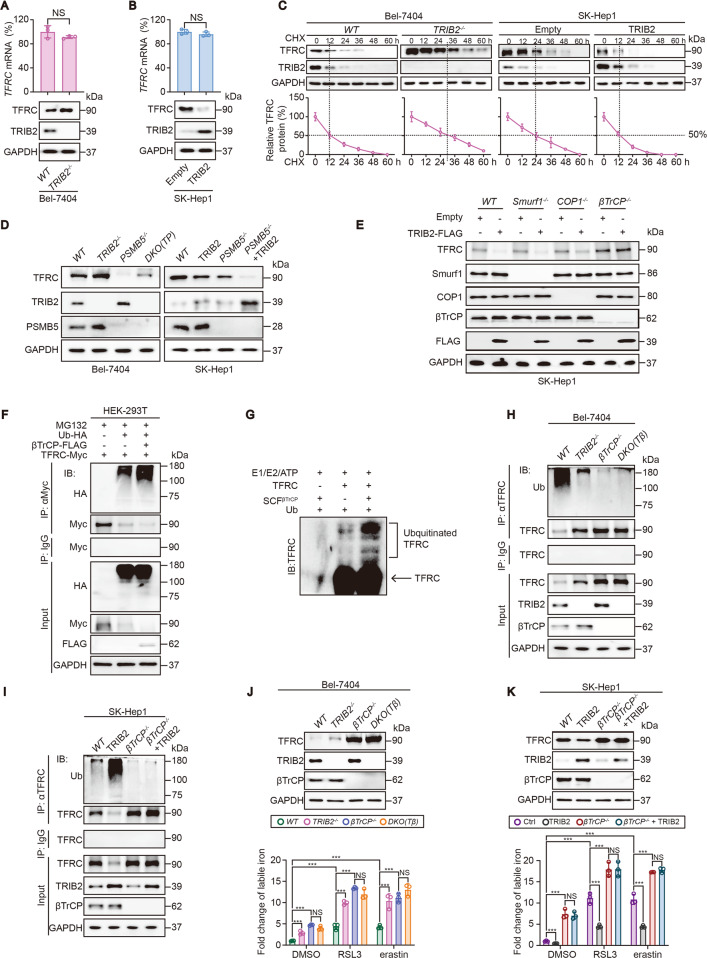
βTrCP-mediated ubiquitination of TFRC is essential for TRIB2 to decline labile iron. **A**, **B** TRIB2 knockout increased while TRIB2 overexpression decreased TFRC expression. TFRC was measured by qPCR and IB in Bel-7404 cells with or without TRIB2 knocked out (**A**) and in SK-Hep1 cells with or without TRIB2 overexpressed (**B**). **C** TRIB2 knockout prolonged while TRIB2 overexpression shortened the half-life of TFRC protein. CHX chase experiments were performed in Bel-7404 cells with or without TRIB2 knocked out and in SK-Hep1 cells with or without TRIB2 overexpressed. **D** TRIB2 regulated TFRC expression in a PSMB5-independent manner. TFRC was measured by IB in Bel-7404 cells with single or double knockout (DKO) of TRIB2 and PSMB5 or in SK-Hep1 cells with or without TRIB2 overexpression, in the presence or absence of PSMB5 knockout. **E** βTrCP but not Smurf1 and COP1 was required for TRIB2 to reduce TFRC in SK-Hep1 cells. TFRC was measured by IB before and after overexpressing TRIB2 in SK-Hep1 cells with or without knockout of Smurf1, COP1, and βTrCP. **F** βTrCP increased the ubiquitination of TFRC. Exogenous Ub-HA, βTrCP-FLAG, and TFRC-Myc were ectopically expressed, as indicated in HEK-293T treating with MG132 (8 µM). The conjugation of TFRC-Myc by Ub-HA was measured by IB using anti-HA antibodies in the immunoprecipitates that immunoprecipitated by anti-Myc antibodies. **G** TFRC could be ubiquitinated by βTrCP, as measured by an in vitro ubiquitination assay using anti-TFRC antibodies after incubation with purified proteins as indicated. **H** TRIB2 deletion-reduced ubiquitination of TFRC was blocked after βTrCP was simultaneously knocked out. Ubiquitination of TFRC was measured by anti-Ub antibodies in the immunoprecipitates that immunoprecipitated by the anti-TFRC antibodies in Bel-7404 cells with single or double knockout of TRIB2 and βTrCP. The immunoprecipitates that pulled down by IgG antibodies were parallel examined by anti-TFRC antibodies. **I** TRIB2 overexpressing-induced ubiquitination of TFRC was diminished after βTrCP was simultaneously knocked out. The measurement for the ubiquitination of TFRC was similarly performed as that in **H**. **J**, **K** TRIB2 altered labile iron was βTrCP-dependent. Expression of TRIB2 and βTrCP and labile iron were measured in Bel-7404 (**J**) and SK-Hep1 cells (**K**) under the treatments as indicated. Data were analyzed by Student’s *t* test and expressed as mean ± SD from three independent experiments. NS non-significance; ***P < 0.001. Images of all the immunoblots are representative of three independent experiments.

Subsequently, we investigated the mechanism by which TRIB2 declines labile iron via TFRC. We found that knockout of TRIB2 upregulated while overexpression of TRIB2 downregulated TFRC protein expression in Bel-7404 and SK-Hep1 cells (Fig. 5A, B). However, neither knockout nor overexpression of TRIB2 affected mRNA level of TFRC in Bel-7404 and SK-Hep1 cells (Fig. 5A, B), suggesting that TRIB2 does not regulate TFRC at mRNA level. Indeed, TRIB2 knockout prolonged while TRIB2 overexperssion shortened the half-life of TFRC protein in Bel-7404 and SK-Hep1 cells (Fig. 5C), indicating that TRIB2 declines TFRC expression via reducing its protein stability. Recently, a new role of TRIB2 has been identified to modulate protein stability via targeting proteasome 20S subunit beta 5 (PSMB5), a component contains major activity site of proteasome [5]. Thereby, we tested whether TRIB2 regulates TFRC protein via PSMB5. Unfortunately, TRIB2 knockout still increased TFRC level when PSMB5 was simultaneously knocked out in Bel-7404 cells (Fig. 5D). Moreover, overexpressing TRIB2-decreased TFRC expression was still observed regardless whether PSMB5 was knocked out or not in SK-Hep1 cells (Fig. 5D). These results demonstrated that TRIB2 declines TFRC expression in a way independent of PSMB5.

The concept that TRIB2 acts as an adaptor protein to enhance the ubiquitination activity of the E3s is well accepted [1]. Furthermore, TRIB2 has been revealed to interact with E3s including Smurf1, COP1, and βTrCP in liver cancer cells [2, 4, 25]. These evidences led us to investigate whether TRIB2 decreases TFRC protein expression via these E3s. As shown in Fig. 5E, only when βTrCP but not Smurf1 and COP1 was knocked out diminished the effects of TRIB2 overexpression to reduce TFRC protein expression in SK-Hep1 cells, demonstrating that TRIB2 regulates TFRC via βTrCP.

To verify whether βTrCP is a genuine E3 of TFRC, we overexpressed βTrCP in HEK-293T cells to examine whether the ubiquitination of TFRC was enhanced under this condition. As expected, TFRC protein expression was decreased accompanied with the enhanced ubiquitination of TFRC following overexpressing βTrCP (Fig. 5F). By in vitro ubiquitination assay, we further confirmed that βTrCP is a genuine E3 for the ubiquitination of TFRC (Fig. 5G). We then investigated whether TRIB2 increases ubiquitination of TFRC and such effects are dependent of βTrCP. In Bel-7404 cells, knocking TRIB2 out resulted in a significant suppression on the ubiquitination of TFRC, and such effects were blocked once upon βTrCP was also knocked out (Fig. 5H). Furthermore, the effects resulted from TRIB2 overexpression to increase ubiquitination of TFRC were also diminished following knocking βTrCP out in SK-Hep1 cells (Fig. 5I). Together, βTrCP is required for TRIB2 to modulate the ubiquitination of TFRC in liver cancer cells.

Because TRIB2 boosts ubiquitination of TFRC via βTrCP, we then examined whether TRIB2-declined labile iron in basal condition and during ferroptosis is also relied on βTrCP. We found that βTrCP knockout significantly elevated basal labile iron level in both Bel-7404 and SK-Hep1 cells (Fig. 5J, K), hinting that the prevented ubiquitination of TFRC enhances iron absorption. We also observed that the opposite outcome on labile iron resulted from knocking out and overexpressing TRIB2 were all diminished once upon βTrCP was knocked out in Bel-7404 and SK-Hep1 cells both in basal condition and following treating with RSL3 and erastin (Fig. 5J, K). Combined with the results from Fig. 4D–G, we concluded that TRIB2 and βTrCP are essential to decline labile iron level via suppressing TFRC both in basal condition and during ferroptosis.

### Both TFRC and βTrCP were critical for TRIB2 to desensitize cells to ferroptosis and reduce ferroptosis-associated lipid ROS generation

**Fig. 6:**
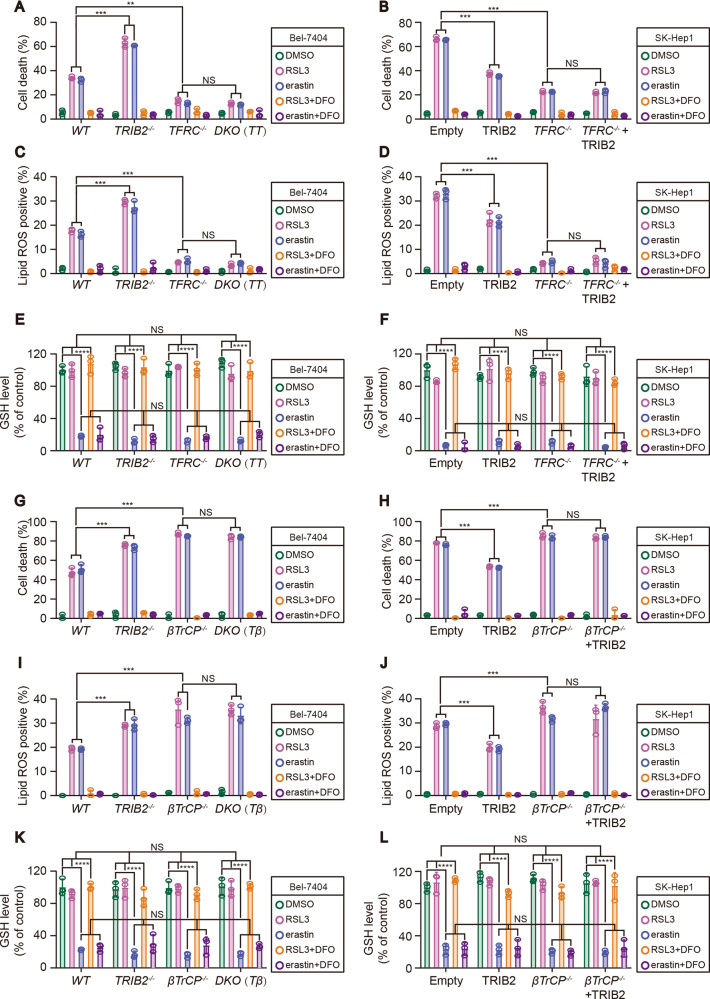
TFRC and βTrCP were both required for TRIB2-desensitized ferroptosis and -reduced ferroptosis-associated lipid ROS generation. Cell death (**A**, **B**), lipid ROS generation (**C**, **D**), and GSH levels (**E**, **F**) were measured by staining with Sytox green (**A**, **B**) and C11-BODIPY (**C**, **D**) following flow cytometry and a GSH assay kit (**E**, **F**), respectively in Bel-7404 and SK-Hep1 cells with or without knockout and overexpression of TRIB2, in the presence or absence of TFRC following treating with RSL3 (5 µM, 12 h) or erastin (10 µM, 12 h) with or without DFO (25 µM, 12 h). Cell death **(G**, **H)** and lipid ROS generation **(I**, **J)** and GSH levels **(K**, **L)** were measured by staining with Sytox green **(G**, **H)** and C11-BODIPY **(I**, **J)** following flow cytometry and a GSH assay kit (**K**, **L**) in Bel-7404 and SK-Hep1 cells with or without knockout and overexpression of TRIB2, in the presence or absence of βTrCP following treating with RSL3 (5 µM, 12 h) or erastin (10 µM, 12 h) with or without DFO (25 µM, 12 h). Data were analyzed by one-way ANOVA and expressed as mean ± SD from three independent experiments. NS non-significance; **P < 0.01; ***P < 0.001.

Then, we assessed whether TFRC and βTrCP contribute to the effects by which TRIB2 desensitizes liver cancer cells to ferroptosis that induced by RSL3 and erastin. We found that the degree of RSL3 and erastin-induced ferroptosis was significantly reduced in *TFRC*^−/−^ Bel-7404 and SK-Hep1 cells (Fig. 6A, B). We also noted that TRIB2 knockout was ineffective to sensitize ferroptosis in *TFRC*^−/−^ knockout Bel-7404 cells (Fig. 6A). Moreover, overexpressing TRIB2 was also unable to desensitize ferroptosis in *TFRC*^−/−^ SK-Hep1 cells (Fig. 6B). Similarly, TRIB2 knockout-strengthened ferroptosis-associated lipid ROS generation was also not observed when TFRC was simultaneously knocked out in Bel-7404 cells (Fig. 6C). Expectedly, the reduced lipid ROS generation resulted from TRIB2 overexpression in SK-Hep1 cells following RSL3 and erastin treatment was not seen upon TFRC was knocked out (Fig. 6D). These results demonstrated that TFRC is essential for TRIB2 to desensitize cells to ferroptosis and reduce ferroptosis-associated lipid ROS generation upon treating with RSL3 and erastin.

Opposite to the effects by knocking TFRC out, the degree by which RSL3 and erastin-induced ferroptosis and ferroptosis-associated lipid ROS generation was significantly higher in *βTrCP*^−/−^ Bel-7404 and SK-Hep1 cells as compared to their parental control cells, this might because βTrCP deficiency increases TFRC stability to elevate labile iron. Also, neither knocking out nor overexpressing TRIB2 influenced sensitivity of the cells to the induction of ferroptosis and ferroptosis-associated lipid ROS generation in *βTrCP*^−/−^ Bel-7404 and SK-Hep1 cells (Fig. 6G–J). Altogether, the effects underlying βTrCP antagonizes TFRC are critical for TRIB2 to desensitize cells to ferroptosis that triggered by RSL3 and erastin.

Reasoning that RSL3 and erastin trigger ferroptosis by targeting GPX4 and system X_C_^−^, respectively, through which finally reduce utilization or production of glutathione (GSH), we thereby tested whether TRIB2/βTrCP/TFRC axis affects sensitivity of liver cancer cells to ferroptosis that induced by RSL3 and erastin via modulating GSH levels. Similar to the prior studies [26], GSH could not be reduced by RSL3, but rather could be significantly suppressed by erastin. Unfortunately, such effects could not be further changed in Bel-7404 and SK-Hep1 cells following manipulation of the expressions of TRIB2, TFRC, or βTrCP (Fig. 6E, F, K, L). These results demonstrated that the TRIB2/βTrCP/TFRC axis influences the sensitivity of liver cancer cells to RSL3- and erastin-induced ferroptosis via a GSH-independent manner.

### TRIB2 exclusively manipulated RSL3- and erastin-induced-ferroptosis independent of GPX4

**Fig. 7:**
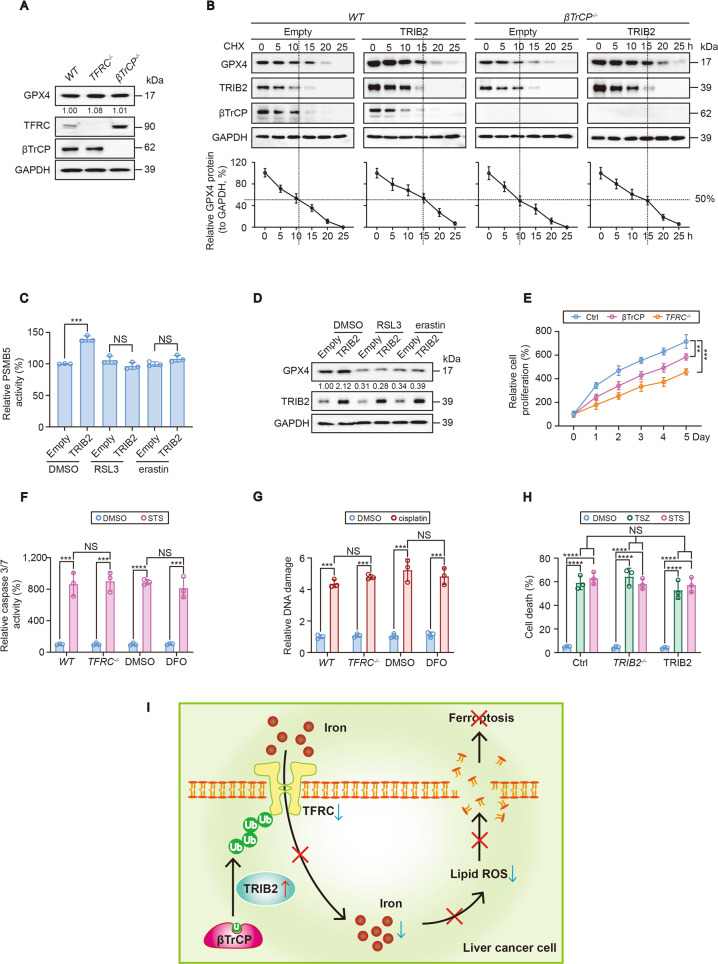
TRIB2 exclusively regulates RSL3- and erastin-induced ferroptosis independent of GPX4 and the schematic presentation of the study. **A** GPX4 in SK-Hep1 cells with or without TFRC or βTrCP knockout. **B** CHX chase of GPX4 with or without TRIB2 overexpression in control cells and SK-Hep1 cells with βTrCP knockout. The relative expression levels of GPX4 were normalized to that of GAPDH and the data from “0 h” were arbitrary set to 100%. **C**, **D** RSL3 and erastin inhibited TRIB2-induced stimulation of PSMB5 activity. PMSB5 activity (**C**) and GPX4 expression (**D**) were measured in SK-Hep1 cells with or without TRIB2 overexpression and treated with or without RSL3 (5 µM, 12 h) or erastin (10 µM, 12 h). **E** Cell proliferation was evaluated by a CCK-8-based method in control cells and SK-Hep1 cells with or without TFRC knockout or βTrCP overexpression. **F** Changes of iron did not influence apoptosis. Caspase 3/7 activities were measured in SK-Hep1 cells with or without knockout of TFRC, or in the presence or absence of DFO (25 µM, 12 h) before further treating with or without STS (100 nM, 6 h). **G** DNA damage was measured by a kit from Abcam in SK-Hep1 cells with or without TFRC knockout or DFO (25 µM, 12 h) administration before further treating with cisplatin (20 µM, 24 h). **H** Cell death was measured by staining with Sytox Green following flow cytometry in control cells and SK-Hep1 cells with or without TRIB2 knockout or overexpression following treating with DMSO, TSZ (1:1000, 4 h) or STS (100 nM, 24 h). **I** Schematic presentation of the study. Briefly, βTrCP-mediated ubiquitination and followed destabilization of TFRC is essential for TRIB2 to decline labile iron in liver cancer cells. Through such mechanism, the sensitivity of cells to ferroptosis is reduced. Data were analyzed by one-way ANOVA and expressed as mean ± SD from three independent experiments. NS non-significance; **P < 0.01; ***P < 0.001; ****P < 0.0001. Images of all the immunoblots are representative of three independent experiments.

TRIB2 stabilizes GPX4 because TRIB2 reduces the available Ub that is essential for the ubiquitination of GPX4 ([5]). As known, GPX4 plays a critical role to protect against ferroptosis [7]. One might speculate that GPX4 is also involved in the roles of TRIB2 to desensitize the cells to ferroptosis. However, the function of TRIB2 to desensitize the cells to RSL3- and erastin-induced ferroptosis seemed merely dependent on TFRC and βTrCP (Fig. 6). Moreover, TFRC and βTrCP were not able to regulate GPX4 and its substrate GSH (Figs. 6 and 7A), and knocking βTrCP out still unable to alter TRIB2-prolonged GPX4 half-life in SK-Hep1 cells (Fig. 7B). These data excluded that βTrCP/TFRC axis manipulates ferroptosis via GPX4. Then, we asked if RSL3 and erastin could block TRIB2-mediated regulation of GPX4 during the process of ferroptosis. Elevating activity of the proteasome PMSB5 subunit is necessary for TRIB2 to maintain GPX4 stability [5]; however, overexpressing TRIB2-resulted elevation of PSMB5 activity was prevented once upon RSL3 and erastin were administrated (Fig. 7C). As a consequence, the upregulation of GPX4 by TRIB2 was also blocked (Fig. 7D). This may further explained why GPX4 is ineffective in the roles of TRIB2 to desensitize cells to ferroptosis that triggered by RSL3 and erastin.

As knocking out TFRC and βTrCP plays contrary roles to control labile iron (Fig. 6), and tumor cells often show iron addiction to sustain cell proliferation [9]; we were curious to know whether TFRC and βTrCP are also essential for cell proliferation in liver cancer cells. Indeed, knocking TFRC out and overexpressing βTrCP both reduced cell proliferation in SK-Hep1 cells (Fig. 7E). However, iron might not be critical for the sensitivity of cells to apoptosis and DNA damage, because neither knocking TFRC out nor administrating with iron chelator DFO changed STS-induced caspase 3/7 activity (Fig. 7F) and cisplatin-induced DNA damage (Fig. 7G).

Finally, we investigated whether TRIB2 exclusively regulates ferroptotic cell death in liver cancer cells. We found that TSZ-induced necroptotsis and STS-induced apoptosis could not be manipulated by either knocking out or overexpressing TRIB2 in SK-Hep1 cells (Fig. 7H). Thus, these results were to some extent, indicative of the exclusive role of TRIB2 to regulate ferroptosis in liver cancer cells.

## Discussion

In the current study, we have demonstrated that TRIB2 has a novel role to desensitize liver cancer cells to ferroptosis, and a declined labile iron level is essential for such a process. Further, we have elucidated that E3 βTrCP-mediated TFRC ubiquitination is important for TRIB2 to reduce labile iron (Fig. 7I). Given that E3, Ub, and TRIB2 are all critical factors that belong to the UPS, patients bearing liver cancer might benefit from ferroptosis-based therapy following appropriate modulation of UPS.

Recently, we have identified the role of TRIB2 to relieve oxidative damage in liver cancer cells via declining the availability of Ub that for the ubiquitination of GPX4 ([5]). Although ROS at low levels might be beneficial by acting as physiological signaling regulators for normal homeostasis, excessive amounts of ROS can cause damage to lipids and eventual ferroptosis [16, 27]. To minimize ROS-incurred over lipid peroxidation, antioxidant defense enzymes are employed [28]. The enzymatic defense system against lipid peroxidation, especially those located at cell membrane consists of antioxidant enzymes, which can detoxify lipid hydroperoxides into their corresponding alcohols [29]. GPX4 plays critical roles in such enzymatic defense process. Knocking out GPX4 in mice results in tissue damage that is associated with the induction of ferroptosis [6]. Inhibition of GPX4 can also selectively kill cancer cells through induction of ferroptosis [6]. Despite TRIB2 stabilizes GPX4, its role to influence ferroptosis sensitivity is not very clear. It is more likely that TRIB2 desensitizes cells to ferroptosis is via a TFRC-dependent manner, and we have also excluded that GPX4 is involved in the roles of TRIB2 to desensitize cells to the RSL3- and erastin-induced ferroptosis. But we still cannot eliminate the involvement of GPX4 in the function of TRIB2 to affect ferroptosis triggered by other agonists. Whether and when the effects by which TRIB2 stabilizes GPX4 and destabilizes TFRC are coordinated or separated to manipulate ferroptosis are still not completely known and needs to be investigated in our future study.

During the investigation of the mechanism underlying how TRIB2 regulates TFRC, we found that E3 βTrCP is essential for TRIB2 to suppress TFRC expression. TRIB2 has the capacity to reduce global Ub [5]; however, TRIB2 is upregulated in liver cancer cells [3]. It seems that the remaining low level of Ub in liver cancer cells is sufficient for βTrCP to ubiquitinate TFRC. The proteasome contains three different catalytically active sites on subunits proteasome 20S subunit beta 1 (PSMB1), subunits proteasome 20S subunit beta 2 (PSMB2), and PSMB5 of the 20S core pore [30]. We previously demonstrated that TRIB2 is capable of reducing global Ub via stimulating PSMB5. In the presence of TRIB2, less Ub is available due to the activation of PSMB5, by which suppresses ubiquitination and followed degradation of GPX4 via PSMB1 and PSMB2 ([5]). We speculate that TFRC is degraded via PSMB5 but not PSMB1 and PSMB2 following ubiquitination, because PSMB5 is stimulated once upon TRIB2 is upregulated [5]; however, the exact mechanism underlying how TFRC is degraded in the proteasome will be discussed in the future. At least in the current study we provided another sample in which UPS is tightly regulated to interfere oxidative stress, such as ferroptosis in liver cancer cells.

Iron metabolism is essential for the occurrence of ferroptosis. Ferroptosis induction leads to ferritinophay [14]. Cargo receptor NCOA4 is required for this process and eventual accumulation of ferroptosis-associated cellular labile iron [24]. However, we found that NCOA4 is not involved in the TRIB2-desensitized ferroptosis, but rather ubiquitination and degradation of TFRC is the key to decline labile iron. Interestingly, we also found that even in the absence of TRIB2, erastin, and RSL3 still lift labile iron level in liver cancer cells (Fig. 4E). Due to the fact that TRIB2 decreases the degree that labile iron is elevated upon ferroptosis, we speculate that ferritinophay is a prerequisite to provide labile iron for the induction of ferroptosis, while TRIB2 and βTrCP-mediated ubiquitination and followed degradation of TFRC determines the sensitivities of liver cancer cells to ferroptosis.

## Conclusion

The effects by which βTrCP-mediated ubiquitination and followed degradation of TFRC to decline labile iron are critical for TRIB2 to desensitize liver cancer cells to ferroptosis. Appropriate reduction of TRIB2 function might be beneficial for patients bearing liver cancer because it will definitely sensitize ferroptosis-based therapy.

## Materials and methods

### Cell culture and vectors

Bel-7404, Bel-7402, SMMC-7721, SK-Hep1, HepG2, and HEK-293T cell lines were obtained from our previous study [31]. Authentication of these cell lines was performed by evaluating short tandem repeat markers, and no mycoplasma contamination was detected. Cells were cultured in DMEM (SH30243, HyClone, Logan, UT, USA) supplemented with 10% fetal bovine serum (Sagecreation, Beijing, China) and 1% penicillin-streptomycin solution (15140122, Gibco, Grand Island, NY, USA). Cells were treated with cycloheximide (CHX, 50 μg/ml, HY-12320, MCE, Shanghai, China), RAS-selective lethal 3 (RSL3, 2 µM, S8155, Selleck), erastin (5 µM, HY-15763, MCE), desferrioxamine (DFO, 25 µM, D9533, Sigma, St. Louis, MO, USA), Staurosporine (STS, HY-15141, MCE), or Necroptosis inducer kit with TSZ (TNF-α, SM-164, and Z-VAD-FMK, C1058S, Beyotime). TRIB2-FLAG-, βTrCP-FLAG-, TRIB2-single guide (sg) RNA-, PSMB5-sgRNA-, Cas9-, Ub-HA-expressing plasmids were generated from our previous studies [4, 5]. TFRC-Myc-expressing plasmids were purchased from Biolink (Shanghai, China). The siRNAs targeting against FTH1, FTL, solute carrier family 40 member 1 (FPN1), TF, TFRC, SLC11A2, and NCOA4 were purchased from GenePharma (Shanghai, China) with the sequences listed in Supplementary Table S1. The plasmids expressing sgRNAs targeting against TFRC, Smurf1, COP1, and βTrCP were constructed by PCR with the primers listed in Supplementary Table S2.

### Immunoblotting (IB)

Conventional protocols were used for IB with the details available elsewhere. The following primary antibodies were used for IB: anti-GPX4 (ab125066, 1:1000), anti-TRIB2 (ab117981, 1:1000), anti-Smurf1 (ab57573, 1:1000), anti-TFRC (ab214039, 1:1000), and anti-COP1 (ab56400, 1:1000) were purchased from Abcam (Cambridge, UK); anti-GAPDH (5174, 1:2000), anti-TFRC (13113, 1:1000), anti-Myc (2276 and 5605, 1:1000), anti-HA (3724 or 2367, 1:1000), anti-FLAG (14793, 1:1000), anti-Ub (3936 or 3933, 1:1000) and anti-βTrCP (4394,1:1000) were purchased from Cell Signaling Technology (CST, Boston, MA, USA); anti-PSMB5 (DF6728, 1:1000) was purchased from Affinity (Changzhou, Jiangsu Province, China). The membranes were incubated with HRP-conjugated secondary antibodies (7076 and 7054, 1:2000, CST) and visualized using Clarity Western ECL substrate (1705060, Bio-Rad, Hercules, CA, USA).

### Quantitative RT-PCR

TRIzol reagent (15596018, Invitrogen, Carlsbad CA, USA) was used to extract total RNA, which was then reverse transcribed into cDNA using a kit from Vazyme (R323-01, Nanjing, Jiangsu, China). All reactions were carried out using a SYBR Green mix (Q711-02, Vazyme). The data from the qRT-PCR were analyzed by the ΔC_t_ method: ΔC_t_ = C_t (target gene)_ − C_t (GAPDH)_, ΔΔC_t_ = ΔC_t (experiment group)_ − ΔC_t (control group)_. The relative expression level for a target gene in the stimulated cells was calculated as follows: relative mRNA level = 2^−ΔΔCt^. The primer sequences are available in Supplementary Table S3.

### Coimmunoprecipitation (co-IP)

Conventional co-IP was performed. The antibodies used in co-IP were anti-Myc (1:200 for IP or 1:1000 for IB, 2276 and 5605, CST), anti-TFRC (1:100 for IP or 1:1000 for IB, 13113, CST or ab214039, Abcam) and anti-IgG (A7028 or A7016, 1:50 for IP, Beyotime, Shanghai, China).

### Flow cytometry

Cells were stained with C11-BODIPY (D3861, 1:1000, Invitrogen) or Sytox green (KGA261, 1:1000, KeyGEN BioTECH, Nanjing, Jiangsu Province, China) and fluorescence-activated cell measuring was performed with BD Canto ll. Data were analyzed with the flow cytometry analysis program FlowJo V10.4.

### Measurement of cell viability

Cells were seeded and adhered to opaque-walled multiwell plates. After specific processing, 100 µl CellTiter-Glo reagent (G7570, Promega, Madison, WI, USA) was added into 100 µl of medium-containing cells. The mixture was mixed for 2 min on an orbital shaker to induce cell lysis and incubated at room temperature for 10 min to stabilize luminescent signal. Synergy microplate reader was used to record luminescence.

### Measurement of AA and AdA in the membrane fraction

Membrane fraction was isolated by the fatty acid extraction kit (MAK174, Sigma) according to the manufacturer’s instructions. The levels of AA and AdA were tested by ELISA-based kits (E-EL-0051, Elabscience and F10423-A, FANKEW, Shanghai, China).

### Measurement of labile iron

Labile iron was tested by an Iron assay kit (MAK025, Sigma) according to the manufacturer’s instructions. Labile iron concentration was calibrated by subtracting the blank value from all readings.

### In vitro ubiquitination assay

E1 (E-306, Boston Biochemistry, Abingdon, Oxfordshire, UK), E2 (E2-603, Boston Biochemistry), Ub (provided by Dr. Lin Jiafei from Shanghai Jiaotong University), TFRC (H00007037-G01, Abnova, Taiwan, China) or SCF^βTrCP^ complex (βTrCP, Cullin1, Skp1, and Rbx1) (Abnova) were incubated at 30 °C for 2 h, and the assay was terminated with protein loading buffer according to the previous study [32].

### Measurement of PSMB5 and caspase 3/7 activities

PSMB5 activity was tested by an Amplit fluorimetric proteasome 20S activity assay kit (AAT-13456, AAT Bioquest, Sunnyvale, CA, USA) and caspase 3/7 activity was tested by a caspase-Glo 3/7 assay system (G8090, Promega) according to the manufacturer’s instructions.

### Measurement of cell proliferation and DNA damage

Cell proliferation and DNA damage were tested by a cell counting kit-8 (CCK-8, HY-K0301, MCE) and DNA damage assay kit (ab211154, Abcam), respectively, according to the manufacturer’s instructions.

### Measurement of reduced GSH

GSH was tested by a GSH assay kit (BC1175, Solarbio, Beijing, China) according to the manufacturer’s instructions. GSH concentration was calibrated by subtracting the blank value from all readings.

### Statistical analysis

Data were presented as mean ± SD. All the data were normally distributed. Sample size was chosen according to previous reports [3, 33]. Tests to examine the differences between groups included Student’s *t* test and one-way ANOVA. A *P* < 0.05 was regarded as statistically significant. Variance was similar between the groups that were being statistically compared.

## Supplementary information

Supplementary table
