# Correction: TRIB2 desensitizes ferroptosis via βTrCP-mediated TFRC ubiquitiantion in liver cancer cells

**DOI:** 10.1038/s41420-021-00597-8

**Published:** 2021-08-06

**Authors:** Susu Guo, Yuxin Chen, Xiangfei Xue, Yueyue Yang, Yikun Wang, Shiyu Qiu, Jiangtao Cui, Xiao Zhang, Lifang Ma, Yongxia Qiao, Jiayi Wang

**Affiliations:** 1 Department of Clinical Laboratory, Shanghai Tenth People’s Hospital, Tongji University, Shanghai, 200072 China; 2 Department of Clinical Laboratory, West China Second University Hospital, Sichuan University, Sichuan, 610041 China; 3 Shanghai Institute of Thoracic Oncology, Shanghai Chest Hospital, Shanghai Jiao Tong University, Shanghai, 200030 China; 4 School of Public Health, Shanghai Jiao Tong University School of Medicine, Shanghai, 200025 China

**Keywords:** Targeted therapies, Oncogenes

Correction to: *Cell Death Discovery* (2021) **7**:196; 10.1038/s41420-021-00574-1, published online 27 July 2021

The original version of this article unfortunately contained a mistake. Due to a typesetting error the following sentence was omitted: “The authors contributed equally: Susu Guo and Yuxin Chen”. We apologize for the error. The original article has been corrected.

