# Supplementary material for: TRIB2 desensitizes ferroptosis via βTrCP-mediated TFRC ubiquitiantion in liver cancer cells
Source: Cell Death Discov. 2021 Jul 27;7:196. doi: 10.1038/s41420-021-00574-1 (PMC8316344; doi:10.1038/s41420-021-00574-1)
Supplement: Supplementary file 1 — Supplementary table [file 41420_2021_574_MOESM1_ESM.doc]

**Supplementary Table S1-3**

**Table S1**

| **Name** | **5'-3'** |
| --- | --- |
| siFTH1 | AUAGAUUUCUGAUUCAUCCCA |
| siFTL | UAAGAAAGCUGCCUAUUGGCU |
| siFPN1 | UCAAAAGGAGGCUGUUUCCAU |
| siPCBP2 | UUGAAGAUGGCAUUAGUGGGU |
| siTF | UGUCUUUGCCAAAAUGUUCCU |
| siTFRC | UAGAGAAUGCUGAUCUAGCUU |
| siSLC11A2 | UUAGAAUAUGAUUCUUACCAG |
| siNCOA4 | AGUUAUCUUUAAUUUGCUGUU |

**Table S1. siRNA sequences for target genes.**

**Table S2Table S2. Primers used for construction of plasmids.**

| **Name** | **5'-3'** |
| --- | --- |
| TFRC-sg-F (lentiCRISPR v2) | CACCGGATCGGTTGGTGCCACTGAA |
| TFRC-sg-R (lentiCRISPR v2) | AAACTTCAGTGGCACCAACCGATCC |
| Smurf1-sg-F (lentiCRISPR v2) | CACCGGATAGCACCGGTGCTGCTGC |
| Smurf1-sg-R (lentiCRISPR v2) | AAACGCAGCAGCACCGGTGCTATCC |
| COP1-sg-R (lentiCRISPR v2) | CACCGGGAGCTGCTGCCTCCTACGC |
| COP1-sg-R (lentiCRISPR v2) | AAACGCGTAGGAGGCAGCAGCTCCC |
| βTrCP-sg-R (lentiCRISPR v2) | CACCGGCGAAGGCATGCTGTCCGCC |
| βTrCP-sg-R (lentiCRISPR v2) | AAACGGCGGACAGCATGCCTTCGCC |

**Table S3. Primers for qPCR.**

| **Table S3** | |
| --- | --- |
| **Name** | **5'-3'** |
| TRIB2-F | GACTCCGAACTTGTCGCATTG |
| TRIB2-R | GGCACGAAAAACGTGGTCT |
| FTH1-F | TCCTACGTTTACCTGTCCATGT |
| FTH1-R | GTTTGTGCAGTTCCAGTAGTGA |
| FTL-F | CAGCCTGGTCAATTTGTACCT |
| FTL-R | GCCAATTCGCGGAAGAAGTG |
| FPN1-F | CTACTTGGGGAGATCGGATGT |
| FPN1-R | CTGGGCCACTTTAAGTCTAGC |
| PCBP2-F | CTTTGGCTGGACCCACTAATG |
| PCBP2-R | CCCTGTACTCTCTCGTATTTCCT |
| TF-F | GTGTGCAGTGTCGGAGCAT |
| TF-R | CATCGGATGGAATGACGCTTT |
| TFRC-F | ACCATTGTCATATACCCGGTTCA |
| TFRC-R | CAATAGCCCAAGTAGCCAATCAT |
| SLC11A2-F | TGGAGATCATGGGGAGTCTG |
| SLC11A2-R | AAGAAAACCTGGTCCGGTGAA |
| NCOA4-F | GAGGTGTAGTGATGCACGGAG |
| NCOA4-R | GACGGCTTATGCAACTGTGAA |
